# Figure of eight suture technique in aortic valve replacement decreases prosthesis-patient mismatch

**DOI:** 10.1186/s13019-023-02260-y

**Published:** 2023-04-10

**Authors:** Nabeel F. Rasheed, Corinne Stonebraker, Zhaozhi Li, Umar Siddiqi, Andy C. H. Lee, Willa Li, Sydney Lupo, Jennifer Cruz, William G. Cohen, Cathy Staub, Daniel Rodgers, Mark Myren, Pamela Combs, Valluvan Jeevanandam, Narutoshi Hibino

**Affiliations:** Department of Surgery, Division of Cardiothoracic Surgery, University of Chicago, 1109 Ashley Lane, Inverness, IL 60010 USA

## Abstract

**Background:**

While the pledget suture technique has been the standard for surgical aortic. valve replacement (AVR), discussion continues regarding the possibility of the nonpledget suture technique to produce superior structural and hemodynamic parameters. This study aims to assess the effectiveness of the figure-of-eight suture technique in AVR, as determined by the incidence of prosthesis-patient mismatch (PPM).

**Methods:**

We reviewed records of patients (N = 629) who underwent a surgical AVR procedure between January 2011 and July 2018 at a single institution. Indexed effective orifice area values and PPM incidence were calculated from implanted valve size and patient body surface area. Incidence of none, moderate, and severe PPM was compared across AVR suture techniques.

**Results:**

A total of 570 pledget and 59 figure-of-eight patients were compared for incidence of PPM. Patients who received AVR with the pledget suture technique had significantly lower echocardiographic measurements of baseline ejection fraction than patients who had received AVR with the figure-of-eight suture technique (p = 0.003). Patients who received the figure-of eight suture had a 14% decrease in moderate PPM compared to patients who received the pledget suture (p = 0.022). Patients who received the figure-of-eight suture also had a significantly higher rate of no PPM (p = 0.044).

**Conclusions:**

The use of the figure-of-eight suture technique in AVR can reduce the incidence of moderate PPM. While the pledget suture is the standard technique in AVR, the figure-of-eight suture technique may offer better structural and hemodynamic outcomes, especially for patients with a smaller aortic annulus.

## Background

Aortic valve replacement (AVR) has evolved into the gold standard of treatment for severe aortic valve disease, with a 3.4% 30-day mortality rate and 3.2% rate of vascular complications [1]. Traditionally, AVR is performed by using interrupted sutures with nonabsorbable pledgets, which are placed under the aortic annular ring to secure the prosthetic valve. The pledget suture technique provides additional reinforcement that is particularly useful in patients with delicate, loose tissue in their aortic ring [2]. With their added support, pledget sutures can further prevent the onset of aortic regurgitation or paravalvular leaks (PVLs) [3]. PVLs follow a generally benign course after AVR, but in their moderate-to-severe forms, they can be a significant risk factor of post-operative mortality [4–6]. Pledget sutures are still met with caution, however, as they may increase the risk of endocarditis, pannus formation, aortic calcification, and reduced effective orifice area (EOA) [4, 7, 8]. Due to these concerns, the interrupted nonpledget suture technique has been considered in AVR, especially in younger patients who do not require significant valvular support. The nonpledget suture technique creates a larger EOA following implantation, which can better accommodate future transcatheter valve replacement (TAVR) and reduce prosthesis-patient mismatch (PPM). PPM describes the disproportion between the EOA of an implanted valve and the patient’s body size, and its presence can significantly impact long-term cardiac and mortality outcomes [14–24].

The pledget and nonpledget suture techniques carry unique differences in design and utility that effect their clinical outcomes in AVR. In comparison to the noneverting pledget technique, Tabata and colleagues found that the simple interrupted suture without pledgets can significantly reduce PPM in small-valve AVR [7]. Kim et al. [8] reproduced these findings in their institution and additionally reported an improved PPM reduction with the figure-of-eight suture technique without pledgets. Despite these results, past reports have found neither suture technique to be correlated with PPM and no significant association between suture type and the incidence of PVLs [9, 10]. Although the nonpledget suture technique has been recognized for its safe design in reducing PPM, current literature on its clinical unitility has remained divided. Additionally, a paucity of studies have investigated outcomes with the figure-of-eight technique despite its potential to reduce PPM in small-valve AVR [8, 13]. We report a retrospective, single-center study to assess PPM in patients undergoing AVR with the pledget or the figure-of-eight suture technique. The findings of this study will contribute to understanding the choice of surgical suture techniques during AVR and elucidate ways to improve outcomes in patients requiring AVR.

## Methods

### Patient selection

From January 2011 to July 2018, 676 patients underwent a surgical AVR using either the pledgeted or figure-of-eight suture technique at The University of Chicago Medical Center. Of the original 676 patients, 629 (93%) met the requisite inclusion criteria. Patients who received a TAVR were excluded. Patients for whom a suture technique other than the pledgeted or figure-of-eight suture was used were excluded. There were three aortic valves for which manufacturer EOA was unavailable: the Edwards Magna sizes 27 and 29, and the St. Jude Trifecta size 19. Patients with unknown implanted valve size, valve manufacturer, manufacturer EOA, preoperative height or preoperative weight were excluded. This study was approved by the Institutional Review Board of The University of Chicago (IRB20-0360) on March 27, 2020. Individual patient consent was waived as the study design is retrospective and there was no interference with patient treatment.

### Surgical technique

**Fig. 1:**
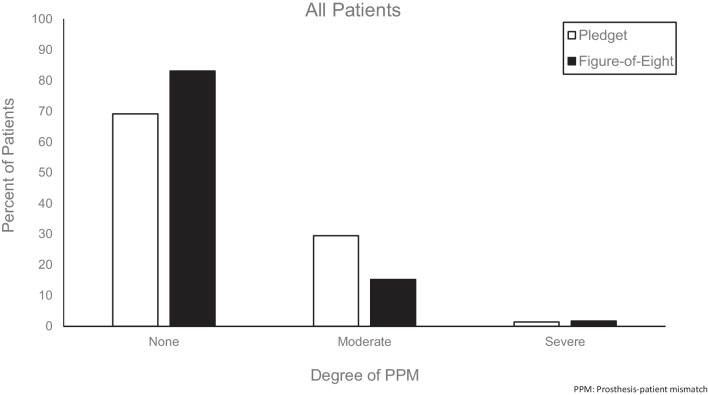
Rate of PPM across AVR suture techniques in the overall patient population. The rates of moderate PPM and no PPM were significantly different between the figure-of-eight and pledget suture technique. Moderate, p = 0.022; Severe, p = 0.59. *PPM* prosthesis-patient mismatch; *AVR* aortic valve replacement

All patients underwent a standard AVR procedure through a complete median sternotomy. The existing aortic valve was de-calcified, and all debris was meticulously removed. In the pledgeted suture technique, 9 to 20 pledgeted mattress 2-0 Ti-Cron™ sutures (Ti-Cron™; Medtronic, Minnesota, USA) were placed around the circumference of the ventricular side of the annulus in a horizontal mattress fashion. Using two needles, all sutures were passed from the ventricular side of the annulus to the aortic side of the annulus and through the prosthetic valve sewing ring. In the figure-of-eight suture technique, 12 to 30 figure of eight 2-0 Ti-Cron™ sutures were used to secure the new aortic valve. One needle was repeatedly passed from the ventricular side of the annulus to the aortic side of the annulus and through the prosthetic valve sewing ring. The other needle remained on the ventricular side of the annulus, achieving semi-intraannular positioning. Both the figure-of-eight and pledget techniques are well visualized in Fig. 1 of the report written by Kim and colleagues [10]. Employing pledgets increases the distance between sutures; therefore, more figure-of-eight sutures could be placed in comparison to the pledget suture technique. All prosthetic valves were properly seated without difficulty.

### Data collection

Using the *Cardio Valve* application (© 2012 Digital Medical Networks), valve EOA was determined. The *Cardio Valve* application utilizes an algorithm derived from existing aortic prosthetic valve literature and valve manufacturer information to estimate EOA for a given valve type and size. Indexed effective orifice area (iEOA) was calculated by dividing the EOA from the *Cardio Valve* application by the preoperative patient body surface area (BSA). PPM was defined according to the iEOA value. PPM for an iEOA greater than 0.85 cm^2^/m^2^ was defined as “none”, for an iEOA between 0.65 and 0.85 cm^2^/m^2^ was defined as “moderate”, and for an iEOA of less than 0.65 cm^2^/m^2^ was defined as “severe”. Incidence of PPM was compared across AVR suture techniques (pledgeted suture technique, figure-of-eight suture technique), valve types (bioprosthetic, mechanical), valve sizes (sizes 19, 21, 23, 25, 27, 29), and valve manufacturers (ATS APex™, Edwards PERIMOUNT™, Edwards Magna™, MCRI On-X™, Medtronic 3F™, Medtronic Mosaic™, St. Jude Trifecta™).

### Statistical analysis

For the statistical analysis, two-sided p values of < 0.05 were considered statistically significant. Continuous data is reported as mean and standard deviation if normally distributed, or median and interquartile range (IQR) if skewed. Continuous variables were compared using Mann–Whitney U test. Categorical variables are expressed as number and percentage of patients and were compared using Fisher’s exact test.

## Results

A total of 629 patients were studied for incidence of moderate and severe PPM. Among suture techniques, 570 patients (90.6%) underwent AVR with pledget sutures and fifty-nine patients (9.4%) underwent AVR with figure-of-eight sutures (Table 1). Eighty-one patients had undergone a prior aortic valve procedure; of these, five patients (6.2%) had undergone a prior aortic valve repair, forty-five (55.6%) had undergone a prior aortic valve replacement, and thirteen (16%) had undergone a prior aortic valvuloplasty. Among valve types, 534 patients (84.9%) received a bioprosthetic valve and 95 patients (15.1%) received a mechanical valve. Among valve manufacturers, 103 patients (16.4%) received an Edwards Magna™ valve, 11 patients (1.7%) received an Edwards PERIMOUNT™ valve, 94 patients (14.9%) received an MCRI On-X™ valve, 415 patients (66.0%) received a St. Jude Trifecta™ valve, and 6 patients (1.0%) received another type of valve (ATS Apex™, Medtronic 3F™, Medtronic Mosaic™).

**Table 1:** Demographic and baseline characteristics between pledgeted suture technique and figure-of-eight suture technique, and p-values

Characteristic	Pledget (N = 570)	Figure-of-eight (N = 59)	p Value
Age	70 (61, 78)	59 (51.5, 67)	< 0.0001
BSA	1.95	2.05	0.008
Female	220 (39%)	18 (31%)	0.26
Smoking status			0.003
Current smoker	63 (11%)	10 (17%)	
Former smoker	150 (26%)	25 (43%)	
Never smoker	355 (63%)	23 (40%)	
DM	182 (32%)	16 (27%)	0.56
HTN	464 (81%)	44 (75%)	0.22
Pre-Op dialysis	51 (9%)	2 (3%)	0.21
Pre-Op creatinine	1.1 (0.9, 1.5)	0.9 (0.8, 1.2)	< 0.001
Prior valve procedure	74 (13%)	7 (12%)	1
Ejection fraction	56 (42, 65)	60 (56.4, 65)	0.003
AV insufficiency			0.02
No	96 (17%)	14 (24%)	
Trace/trivial	84 (15%)	8 (14%)	
Mild	131 (23%)	12 (21%)	
Moderate	144 (26%)	6 (10%)	
Severe	105 (19%)	18 (31%)	
AV stenosis			0.39
No	200 (36%)	24 (42%)	
Yes	358 (64%)	33 (58%)	
CPB time	161 (117, 215)	137 (108, 175)	0.014
Total LOS	10 (7, 18)	8 (6, 12)	0.003
LOS surgery to discharge	8 (6, 14)	7 (5, 9.5)	0.006

*BSA* body surface area; *AV* aortic valve; *DM* diabetes mellitus; *HTN* hypertension; *CPB* cardiopulmonary bypass; *LOS* length of stay (in hospital after aortic valve replacement)

Demographic characteristics, medical history and other pre-, peri-, and post-operative patient data were compared between the pledgeted and figure-of-eight patients (Table 1). There was no significant difference between groups in terms of sex, history of diabetes mellitus, preoperative hemodialysis, or past aortic valve surgery. The body surface area measurements of the figure-of-eight group was significantly higher than the pledget group (p = 0.008). Additionally, there was no significant difference between groups in baseline measurements of aortic valve stenosis. Patients who received a figure-of-eight suture were more likely to be former or current smokers and were more likely to have severe aortic valve insufficiency at baseline. Figure-of-eight suture patients also had a higher average baseline ejection fraction. Patients who received a pledgeted suture were more likely to be older, had a higher average baseline creatinine level, spent more time on cardiopulmonary bypass, and had a longer average hospital length of stay, both total and postoperatively.

Patients who received the figure-of-eight suture exhibited significantly lower rates of moderate PPM compared to patients who received pledget sutured (Fig. 1; p = 0.022). The pledget suture technique further remained independently associated with moderate PPM when adjusting for valve type and manufacturer: OR 2.65 (CI 1.35–4.70, p = 0.007). Of the 570 pledgeted suture patients, 167 exhibited moderate PPM (29.3%), compared to 9 out of 59 figure-of-eight suture patients (15.3%). Patients who received the figure-of-eight suture also more commonly had no PPM (p = 0.044). Of the 570 pledgeted suture patients, 394 demonstrated no PPM (69.2%), compared to 49 out of 59 figure-of-eight suture patients (83.1%).

The association between suture technique and PPM was further compared between small (19–23 mm) and large (25–29 mm) valve sizes (Table 2). In the small valve cohort, the rate of moderate PPM in patients who received the pledgeted suture was higher than that of the figure-of-eight technique. However, this result was not statistically significant due to a limited sample size of small-valve patients (p = 0.14). In patients who were implanted with larger valves, there was neither a statistically significant nor a practically significant difference in PPM among suture techniques.

**Table 2:** Rate of PPM across AVR suture techniques and aortic valve sizes.

Valve size	Pledget (N = 570)	Figure-of-eight (N = 59)	p Value
*Small, 19–23 mm*			
Number of patients	299	18	
iEOA, cm^2^/m^2^	0.86 (0.77, 0.98)	0.89 (0.82, 0.97)	0.3
Moderate PPM	141 (47.2%)	5 (27.8%)	0.14
Severe PPM	8 (2.7%)	1 (5.6%)	0.41
*Large, 25–29 mm*			
Number of patients	271	41	
iEOA, cm^2^/m^2^	1.03 (0.93, 1.15)	1.05 (0.92, 1.15)	0.77
Moderate PPM	27 (9.9%)	4 (9.8%)	1
Severe PPM	0 (0%)	0 (0%)	1

Patients were divided into two cohorts, small (valve sizes 19–23) and large (valve sizes 25–29)

Values are denoted as either *number of* patients (%) or *median* (IQR).

*PPM* prosthesis-patient mismatch; *AVR* aortic valve replacement

## Discussion

Patients undergoing the figure-of-eight suture technique had a significantly lower rate of moderate PPM in comparison to the pledgeted cohort (Fig. 1). Tabata and colleagues achieved similar results and found their pledgeted cohort to have a higher incidence of PPM than their nonpledgeted group [9]. Unlike our methodology, however, they employed a simple interrupted suture as their nonpledgeted technique. To our knowledge, Kim et al. have written the only other report to compare PPM outcomes in patients receiving the pledget and figure-of-eight suture [10]. Notably, they study a sample with an East Asian demographic, whereas we study Western patients who generally require larger replacement valves and have different hemodynamic outcomes compared to their Eastern counterparts [11, 12]. Additionally, they focus on comparing suture techniques employed in a small aortic annulus. We assess patients with both a small and large aortic annulus. They report that both the pledget suture technique and figure-of-eight suture technique were associated with moderate PPM. However, they combined their pledget and figure-of-eight cohorts into one sample for univariate and multivariate analysis. As a result, their pledget and nonpledget techniques were assessed together as a single risk factor for PPM incidence. Ugar and associates conducted a one-year retrospective analysis, investigating hemodynamic data in particularly Western patients [11]. They did not vary suture technique in their pledgeted cohort, and likewise, found no association between the noneverting pledget-reinforced suture and the incidence of severe PPM. In contrast with our study, however, they compared these pledget outcomes with the everting mattress suture technique.

Most recently, Saisho and colleagues investigated suture technique in AVR with an ex vivo model and achieved a significantly larger EOA with the figure-of-eight technique compared to the pledget technique [13]. Their model included a porcine aortic root, the PERIMOUNT Magna Ease 21 mm valve, and a mock circulation loop that simulated physiologic flow and captured EOA and leakage volume. The larger EOA from the figure-of-eight technique was credited to its intra-annular valve positioning and orientation of its sutures, which is consistent with our findings. Since one suture enters from the supra-annular end and the other passes through the ventricular side, the tissue can be ultimately pulled outwards from the valve orifice. In contrast, the pledget technique grants supra-annular valve positioning and its suture orientation can cause tissue to protrude through the left ventricular outflow tract and obstruct flow.

We utilized Fischer’s exact test to compare the rates of moderate PPM by procedure type (pledget vs figure-of-eight). Dividing the patients into those with smaller and larger valve sizes reduced the sample size, lowering statistical power. However, a significant difference in moderate PPM was observed when the valve size groups were combined, likely due to the larger sample size and greater power. The small valve size group in particular did not reach statistical significance despite a large numerical difference in the proportion of patients with moderate PPM, possibly due to the small amount of figure-of-eight patients. Consideration of Simpson’s paradox due to the larger fraction of figure-of-eight patients who were of large valve size, which possessed a lower rate of PPM, are important, but cannot be resolved given the sample size limitations of the present study.

We chose to evaluate PPM, as it is a well-known, modifiable predictor of post-operative hemodynamic abnormalities and all-cause mortality [14–24]. This may be due to the high-pressure gradients caused by PPM, which can lead to left-ventricular hypertrophy and ultimately result in left ventricular failure. In a systematic review and meta-analysis of 34 observational studies, Head et al. found moderate and severe PPM to significantly predict all-cause mortality and cardiac-related mortality [22]. Tasca and colleagues reached similar findings between PPM and cardiac-related mortality, and further characterized PPM as a predictor of mid-term mortality [23]. However, there remain several studies that report a small impact of PPM on mortality and adverse hemodynamic outcomes [24–26]. These studies define PPM as a product of the internal geometric orifice area (GOA) rather than the EOA of the prosthesis. Although this measurement is more reproducible than the EOA, utilizing the GOA has been shown to be a poor predictor of transvalvular pressure gradients following AVR [24, 27]. Thus, these studies cannot be drawn upon to create an accurate relationship between PPM and post-operative mortality or sequalae.

In addition to PPM, several other complications can occur following AVR and increase mortality risk, including infectious endocarditis, chronic lung disease, and reduced left ventricular ejection fraction [8]. Factors such as age, gender, and coronary artery disease can further compound this risk; however, these complications and factors cannot be readily prevented or manipulated. Although this information may offer some insight regarding AVR risk for patients, it cannot be used to develop a strategy for reducing AVR mortality risk. In this present study, we assert that suture design can be modified by surgeons to limit PPM; particularly, the nonpledgeted figure-of-eight suture method can be used as a prospective strategy for reducing post-AVR mortality risk and hemodynamic abnormalities in patients with small valve sizes.

TAVR has gained traction as a beneficial, minimally invasive procedure in patients who are deemed inoperable or who are opposed to surgical AVR. According to Puri et. al., TAVR grants equivalent clinical outcomes to surgical aortic valve replacement and maintains clinical superiority in patients with severe aortic stenosis [28]. However, the degree of benefit following TAVR depends heavily on trans-aortic pressure gradients, and for a large number of patients with low-pressure gradients, TAVR has minimal functional benefit post-operatively [29–31]. In a cohort undergoing TAVR for severe aortic stenosis, Yadav and colleagues report that each 10 mmHg decrease in transaortic pressure gradients results in a 20% increase in mortality [29]. PPM is a measure of iEOA and has a strong association with low transaortic pressure gradients. Considering the figure-of-eight suture technique yields low rates of moderate-to-no PPM, this method may also minimize adverse outcomes for patients undergoing TAVR who have critical aortic stenosis. The figure-of-eight technique can further reduce the incidence of annular rigidity and calcification, adding to its reliability in TAVR procedures.

Regarding the limitations of our study, a relatively small cohort of patients at our center underwent AVR utilizing a figure-of-eight suture. This was due to the restricted, retrospective nature of our study and the general low incidence of figure-of-eight sutures utilized in AVR procedures. As a result, we did not divide our sample into smaller cohorts based on valve type and manufacturer; however, we employed logistic regression to confirm that these variables were not confounders. The pledget suture technique remained independently associated with moderate PPM after adjusting for valve type and manufacturer. Regarding suture technique, there was variability in the number of pledgeted sutures employed by each surgeon, and a lower number of pledget sutures may narrow the aortic root and result in PVLs. However, the majority of patients in the pledget cohort received 12–15 sutures (71.2%), whereas 18 patients received 9–11 sutures (3.2%) and 36 patients received 16–23 sutures (6.3%). Other surgeons did not comment on the number of pledgeted sutures utilized during their AVR operations (19.3%). We were unable to control for this variability due to the retrospective design of our study and differences in surgeon preference; however, a small subset (3.2%) of our patient population received below 12 pledget sutures, which could have caused a narrowing of the aortic root. We further did not associate suture technique with direct clinical outcomes such as paravalvular leaks and ejection fraction, as these comparisons have already been conducted in past literature [3, 12]. PPM is also a strong predictor of mortality and hemodynamic outcomes, so we can imply from our findings that suture type has an indirect yet significant influence on these clinical measures. Another limitation is our use of the cardiovalve application to estimate EOA values. This was necessited by the usage of past echocardiograms which did not include EOA measurements. As a result, we could not directly assess EOA in our patients, and instead utilized a reliable application to conduct indirect estimates.

## Conclusion

Suture technique can have a significant impact on PPM and its post-operative outcomes following AVR. Physicians operating AVR have mainly limited their approach to the simple interrupted suture technique or the interrupted suture with unabsorbable pledgets. We study a far-less utilized method, the figure-of-eight suture, and present it for the first time as a solution that can reduce moderate PPM following AVR. We also report that the figure-of-eight suture can yield significant rates of no PPM and offset adverse hemodynamic outcomes. Although the pledget suture has long been considered the standard technique in AVR, the figure-of-eight suture method may grant superior structural and hemodynamic results, especially in patients with a small aortic annulus who are at greater risk for such mismatch.

## Data Availability

The dataset supporting the conclusions of this article is available in the University of Chicago electric medical record repository.

## Abbreivations

AVR: Aortic valve replacement
PVL: Paravalvular leak
EOA: Effective orifice area
iEOA: Indexed effective orifice area
TAVR: Transcatheter valve replacement
PPM: Prosthesis-patient mismatch
BSA: Body surface area
IQR: Interquartile range
GOA: Geometric orifice area
